# The Role of MicroRNAs in Hepatoblastoma Tumors

**DOI:** 10.3390/cancers11030409

**Published:** 2019-03-22

**Authors:** Ion Cristóbal, Marta Sanz-Álvarez, Melani Luque, Cristina Caramés, Federico Rojo, Jesús García-Foncillas

**Affiliations:** 1 Cancer Unit for Research on Novel Therapeutic Targets, Oncohealth Institute, IIS-Fundación Jiménez Díaz-UAM, E-28040 Madrid, Spain; 2 Translational Oncology Division, Oncohealth Institute, IIS-Fundación Jiménez Díaz-UAM, E-28040 Madrid, Spain; 3 Pathology Department, IIS-Fundación Jiménez Díaz-UAM, E-28040 Madrid, Spain; marta.sanza@quironsalud.es (M.S.-Á.); melani.luque@quironsalud.es (M.L.); frojo@fjd.es (F.R.); 4 Medical Oncology Department, University Hospital “Fundación Jiménez Díaz”, UAM, E-28040 Madrid, Spain

**Keywords:** microRNA, signaling, therapy, biomarkers, hepatoblastoma

## Abstract

Hepatoblastoma is the most common hepatic malignancy during childhood. However, little is still known about the molecular mechanisms that govern the development of this disease. This review is focused on the recent advances regarding the study of microRNAs in hepatoblastoma and their substantial contribution to improv our knowledge of the pathogenesis of this disease. We show here that miRNAs represent valuable tools to identify signaling pathways involved in hepatoblastoma progression as well as useful biomarkers and novel molecular targets to develop alternative therapeutic strategies in this disease.

## 1. Introduction

Hepatoblastoma (HB) is a pediatric tumor that arises from hepatic progenitors or hepatoblasts. It is the most common malignancy of the liver occurring in the pediatric population, with an annual incidence rate of 1.5 cases per million that represents around 1% of total cancers in childhood [1]. However, its incidence has increased by 2.7% per year over the last decades, probably due to the improved survival of premature infants [2]. Disease stage is the current key determinant of patient outcome, and the subgroup of cases with lower risk has a 5-year event-free survival rate of 80% that decreases to 30–40% in the subgroup of high risk or after relapse [3]. Progressive advances in surgical techniques and chemotherapy regimens have resulted in higher survival rates in cases with localized disease, but in that 25% of cases who develop metastases, the prognosis remains very poor [4]. Although the Wnt/β-catenin, Myc, and Hippo pathways have been reported to be involved in the pathogenesis of HB tumors, little is still known about the molecular basis of this disease. In fact, deletions and missense mutations affecting β-catenin protein that cannot be degraded have been found in more than 80% of HB cases, further highlighting the role of this pathway in the disease development [5,6,7]. Moreover, deregulation of the Hippo pathway promotes proliferation, migration and apoptosis resistance, and there has been reported interesting crosstalk between Hippo signaling and microRNAs (miRNAs) in cancer progression that could be of high relevance in HB tumors [8]. Therefore, it is urgent to identify novel targets and signaling pathways altered in this disease in order to develop more effective therapeutic strategies to improve HB patient outcomes.

**Figure 1:**
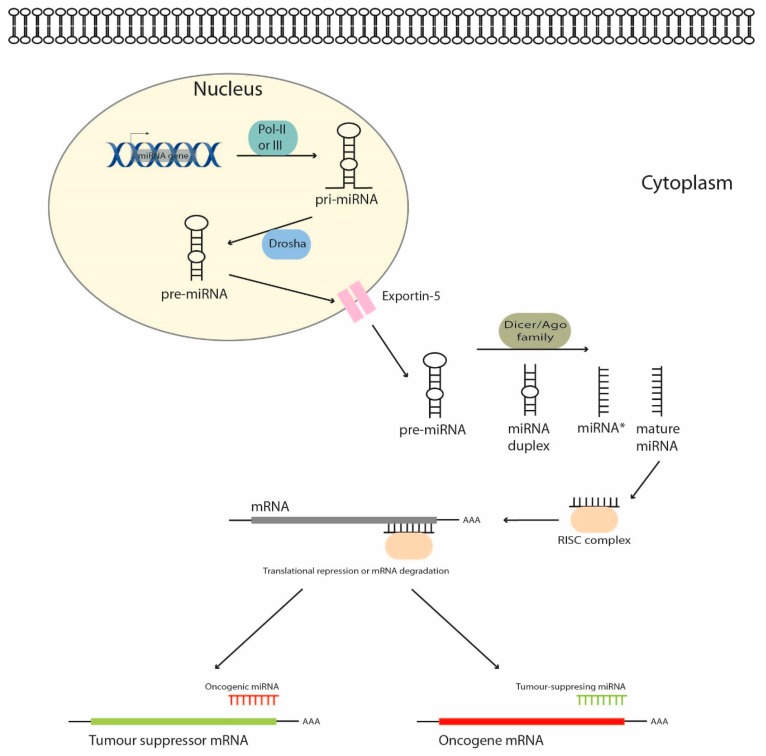
MicroRNA biogenesis in mammalian cells. MiRNA genes are transcribed by RNA polymerase to form the immature pri-miRNA, which is cut by Drosha to liberate a pre-miR which is transported by Exportin-5 from the nucleus to the cytoplasm. The pre-miR is then cleaved by Dicer and the mature miR binds to the RNA-induced silencing complex (RISC) and targets a specific mRNA.

MiRNAs are a class of small (19–25 nucleotides) single-stranded and non-coding RNAs that serve as major regulators of gene expression through their ability to bind and post-transcriptionally inhibit the expression of specific target messenger RNAs [9]. The microRNA biogenesis and functional role are schematized in Figure 1. In the nucleus, miRNA genes are transcribed by RNA polymerase II (or less frequently polymerase III) to form pri-miRNAs, which include hundreds of nucleotides and a terminal loop structure with flanking segments. Next, Drosha RNase III endonuclease cuts the pri-miRNA to liberate a pre-miRNA hairpin (a 60–70 nucleotide long hairpin RNA with 2 nucleotides overhangs at its 3′-end). The pre-miRNA is then transported by exportin-5 from the nucleus to the cytoplasm. The pre-miRNA duplex contains the mature miRNA guide and its complementary passenger strand (miRNA *, *: complementary passenger strand). Considering the widely accepted model, miRNA * is degraded, although evidence suggests that miRNA * may also be functional as well. In the cytoplasm, the pre-miRNA is cleaved by Dicer RNase III endonuclease into a single-strand mature miRNA that binds to proteins of the Argonaute (Ago) family, assembling the RNA-induced silencing complex (RISC). Finally, the “seed region” (a 5′-end region of the mature miRNA, which includes 2–8 nt) binds the 3′-UTR of the target mRNA (Figure 1). According to the match level, the target mRNA will be degraded or blocked, and its translation inhibited, but without decreasing its expression levels. In fact, the complementarity in most animal miRNAs is imperfect, which results in translation inhibition [10]. Of note, each miRNA is able to regulate up hundreds of target genes, affecting the activity of entire signaling pathways. On the contrary, a specific gene is often regulated by a high number of different miRNAs and can have up to 50 miRNA binding sites [11]. Thus, miRNAs are predicted to regulate approximately 60% of the human genes [12]. 

Of importance, miRNAs play a key role in regulating processes like proliferation, differentiation, apoptosis, invasion, angiogenesis, and metastasis via gene expression manipulation [9]. In 2002, the first report about the role of miRNAs in cancer established that the gene cluster containing the miRNAs miR-15 and miR-16 is deleted in most people with chronic lymphocytic leukemia [13]. Further studies have shown that miR-15 and miR-16 act as tumor suppressors by targeting the *BCL2* oncogene, which is involved in cell survival [14]. The importance of miRNAs in human cancer and their potential clinical impact have been progressively growing. In fact, in 2014 the first phase-I clinical trial that used miRNAs as a tool for cancer therapeutics started [15]. 

MiRNAs expression is frequently deregulated in many tumors and their specific target genes determine whether they act as tumor suppressors or oncogenes in human cancers [9]. Thus, the upregulation of oncogenic miRNAs reduces expression of tumor-suppressor proteins, but downregulation of tumor suppressor miRNAs results in increased levels of oncogenic proteins. Modulation of miRNA expression is thought to be an important mechanism by which tumor suppressor proteins and oncoproteins exert some of their effects. Thus, the c-MYC oncoprotein induces the expression of the miR-17-92 cluster [16] and also represses the transcription of many miRNAs with tumor suppressor roles, such as the let-7 family [17]. In concordance, reduced expression of let-7 miRNAs has been reported in many cancers and correlates with poor outcomes [9]. The let-7 family targets RAS proto-oncogene, GTPase (RAS), which is involved in the regulation of essential processes such as cell growth, differentiation, or survival [18]. Moreover, the tumor suppressor p53 induces the expression of the miR-34 family in response to DNA damage and loss of miR-34a expression is associated with metastasis and recurrence of prostate cancer [19], and miR-34b and miR-34c expression is lost through different molecular mechanisms including deletion, hypermethylation, or downregulation in 90% of colorectal cancers [20]. Furthermore, negative regulation of specific targets genes is not the only method by which miRs are implicated in human cancer. Mutations affecting miRs binding sites in the 3’untranslated region (UTR) of oncogenes are correlated with an increased risk of cancer. For instance, a single nucleotide polymorphism in the 3′UTR of the *KRAS* oncogene has been found to significantly increase the risk of non-small-cell lung cancer [21]. Some miRs are also involved in stemness regulation such as miR-296, miR-134, miR-470, and the miR-34 family, which targets genes essential for pluripotency as *Oct4*, *NANOG*, *SOX2*, *NOTCH*, and *BCL2* [22]. Finally, miRs are important for regulating angiogenesis. The activation of proliferation and migration pathways in vascular smooth muscle cells are key steps to promote angiogenesis by tumor cells. Thus, in nasopharyngeal carcinoma cells, the miR-15a/16-1 cluster controls angiogenesis by targeting the angiogenic factors *VEGFA* and *MET* [23].

## 2. MicroRNAs as Regulators of Signaling Pathways in Hepatoblastoma Cells

**Figure 2:**
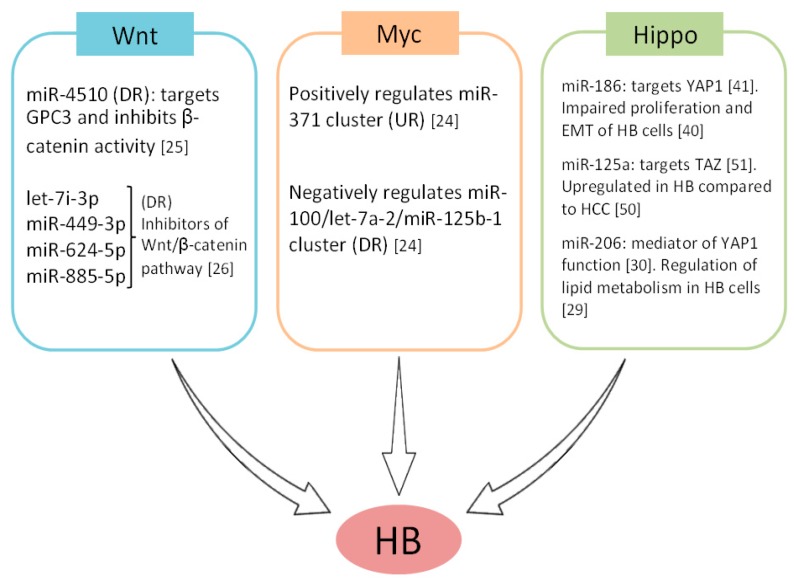
MicroRNAs participating in the key signaling pathways involved in the pathogenesis of hepatoblastoma (HB) tumors. DR: downregulated; UR: upregulated.

The oncogenic effects derived from the activation of both Myc and Wnt pathways, alterations which have been described to play key roles in HB pathogenesis, are mediated at least in part by miRs. Thus, undifferentiated aggressive HB showed the expression of miR-100/let-7a-2/miR-125b-1 and miR-371-3 clusters downregulated and overexpressed respectively. Both clusters were found to be regulated by Myc in an opposite manner and contributed to generate an HB aggressive phenotype regulating proliferation and tumorigenicity in vivo [24]. Moreover, miR-4510 functions as a tumor suppressor since it directly targets the glypican-3 (GPC3) oncogene and impairs the Wnt/β-catenin pathway. In fact, miR-4510 inhibits the transcriptional activity of β-catenin without affecting its expression, decreasing HuH6 cell viability [25]. Moreover, let-7i-3p, miR-449-3p, miR-624-5p and miR-885-5p have also been identified as regulators of β-catenin that inhibit Wnt signaling in HB [26] (Figure 2).

However, several works exploring the role of miRs in HB have progressively provided additional pathways altered in this disease. In vitro studies using the HB cell line HepG2 has shown that the ATP-binding cassette transporter A1 (ABCA1) is a target of miR-101, and that this miR is overexpressed by IL-6 and TNF-α, thereby inhibiting cholesterol efflux. Thus, high levels of miR-101 result in cholesterol retention under inflammatory conditions [27]. More recently, other studies using the same cell line HepG2 identified the efflux transporter ABCG2 as a direct target of miR-655-3p [28], and miR-206 was found involved in the regulation of lipid metabolism [29]. Of note, miR-206 has been reported to mediate YAP1 function, which could be of high relevance in HB [30]. In addition, the GATA4/miR-125b/DKK3 signaling axis has emerged as a major regulator of cell proliferation, migration, and invasion. The transcription factor GATA4 plays an oncogenic role through its binding to the miR-125 promoter which inhibits its transcription, thereby impairing the suppression of the miR-125b target DKK3 [31]. Moreover, the miR-492, described as overexpressed in metastatic HBs [32], has been recently reported to play an oncogenic function in HB enhancing proliferation, anchorage-independent growth, migration, and invasion, further straightening its previously proposed role in HB progression. Moreover, CD44 was identified as a direct target of miR-492 also involved in metastatic progression [33]. Considering the previously described tumor suppressor role of miR-124 in both cholangiocarcinoma [34] and HCC cells [35], Wang and colleagues performed a systematic analysis to determine the potential role of this miR and its molecular mechanism in HB. HepG2 cells were transfected with a miR-124 precursor and differentially-expressed genes were screened at different times by microarray analyses. A miR-124-target mRNA network and pathway enrichment analyses were carried out, observing significant changes in small GTPase-mediated and Ras protein signal transduction together with regulation of actin cytoskeleton, D-glutamine and D-glutamate and axon guidance pathways [36]. Furthermore, it has been demonstrated that the tumor suppressor miR-26a-5p directly targets LIN28B and AURKA in HB cells, which highlights the relevance of the LIN28B-RAN-AURKA axis in the pathogenesis of this disease and the role of miR-26a-5p as a repressor of this signaling [37].

Finally, the role of long non-coding RNAs (lncRNAs) in HB has been explored in several studies. It has been reported that a signaling network involving miR-34a-5p, the lncRNA TUG1 and VEGF plays a relevant role in angiogenesis and HB progression, and in which lncRNA-TUG1 functions as a miR-34a sponge regulating the expression of its target VEGF [38]. Recently, the lncRNA NEAT1 has also been described as a relevant mediator of HB pathogenesis. This lncRNA exerts its function by inhibiting miR-129-5p, which promotes migration and invasion as well as epithelial to mesenchymal transition by modulating E-cadherin and N-cadherin levels [39]. Moreover, the lncRNA OIP5-AS1 inhibits miR-186a-5p-mediated suppression of ZEB1, which promotes the proliferation and EMT of HB cells [40]. Of relevance, miR-186 has been reported to target YAP1, thereby inhibiting the Hippo pathway in HCC, which could have important implications also in HB tumors [41], since YAP1 has been reported to play a relevant role in the pathogenesis of this disease [42,43,44].

## 3. MicroRNAs as Potential Therapeutic Targets

The recent advances in our knowledge about the relevant role that miRs play in HB have highlighted their potential efficacy as novel molecular targets in this disease (Table 1). Thus, an in vivo cooperation between the miR-100/let-7a-2/miR-125b-1 and miR-371-3 clusters in HB has been demonstrated, and the overexpression of the first cluster delayed tumor occurrence, whereas the simultaneous inhibition of the miR-371 cluster totally blocked tumor formation [24]. Moreover, miR-122 has been proposed as a potential target in HB based on its ability to sensitize cells to 5-FU via Bcl-2 and Bcl-XL downregulation, and P53 activation. However, the authors performed their experimentation using the hepatocellular carcinoma cell line BEL-7402/5-FU and proper validation using HB models would be needed [45]. The mitochondrial uncoupling protein 2 (UCP2) has been identified as a target of miR-214 in the hepatoblastoma cell line HuH6. Of importance, UCP2 overexpression mediates resistance to gemcitabine in HCC patients. Moreover, ectopic miR-214 expression markedly decreased HuH6 cell viability [46]. These results are in concordance with previous observations indicating lower miR-214 levels in HB samples than in non-tumor tissues [47], highlighting the potential therapeutic value.

As the Wnt/β-catenin signaling is a main therapeutic target in HB, several studies have explored the role of miRs in this pathway. Thus, Indersie and colleagues identified 4 miRs (let-7i-3p, miR-449-3p, miR-624-5p and miR-885-5p) decreased in HB samples compared to normal liver controls that regulate β-catenin expression and inhibit both cell proliferation and Wnt/β-catenin activity in vitro. Moreover, miR-624-5p impaired tumor growth in vivo and directly targeted β-catenin by its 3′UTR binding [26]. Another study showed that transfection with the miR-4510 precursor resulted in decreased cell proliferation and induced apoptosis of HuH6 cells probably through the inhibition of the Wnt/β-catenin pathway, indicating the potential usefulness of this miR as a therapeutic target [25].

Furthermore, several works have demonstrated that miR-34a, miR-125b, miR-492, miR-378a, and miR-26a-5p could represent novel valuable molecular targets in HB. In fact, transfection with a precursor of the miR-34a-5p has been shown to markedly reduce HB tumor growth in vivo together with lower microvascular density and number of proliferating tumor cells, which further highlights the role of this miR in tumor angiogenesis [38]. The GATA4/miR-125b/DKK3 axis has been proposed as a potential therapeutic target in this disease. Specifically, inhibition of miR-125b promoted cell growth, migration, and invasion of hepatoma HuH6 cells [31]. The oncogenic miR-492 has been recently proposed as a potential therapeutic target based on its ability to regulate the metastatic progression of HB via CD44 regulation [33]. It has been reported that miR-378a inhibits proliferation and invasion abilities as well as enhances the sensitivity of HB cells to sorafenib-based chemotherapy through the negative regulation of its targets VEGFR, PDGFRβ, and c-Raf [48]. In addition, the tumor suppressor miR-26a-5p inhibits cell proliferation and colony formation of HepG2 and HuH6 cells, suggesting its potential usefulness as a novel molecular target in HB [37].

Interestingly, the role of circular RNAs (circRNAs) as potential miR regulators has been investigated in a study that performed circRNA microarrays in HB samples and normal liver controls. The authors found that circ_0015756 acts as an miR-1250-3p sponge, inhibiting its tumor suppressor function and affecting cell viability and invasion of HuH6 cells [49].

## 4. MicroRNA Expression Patterns and Their Potential Clinical Impact as Novel Biomarkers in Hepatoblastoma

Apart from being essential for all the tumor-related processes mentioned above, miRs expression profiles have progressively emerged as useful biomarkers in human cancers. In fact, miR expression profiling in HB has been analyzed in several studies [24,32,44,50]. The first study involving the analysis of miR expression in HB patients was performed by Magrelli and colleagues in 2009. MiR expression was assayed by microarray and quantitative reverse transcription polymerase chain reaction in samples from nine HB cases. They described a set of 13 miRs which were able to discriminate tumor from non-tumor tissues and 5 miRs differentially expressed in HB compared to hepatocellular carcinoma (HCC) (four significantly upregulated: miR-214, miR-199a, miR-150 and miR-125a, and one downregulated: miR-148a) [50]. Of interest, miR-125a has been reported to target Tafazzin (TAZ), thereby impairing retinoblastoma proliferation, and it would be very interesting to analyze its potential role in regulating the Hippo pathway in HB [51]. Next, distinct miR profiles were observed between mild and aggressive HB subtypes with 20 miRs differentially expressed (5 miRs upregulated and 15 downregulated in the aggressive subtype in comparison with the mild subtype). Of importance, a four-miR signature (miR-100, let-7a, miR-371, and miR-373) efficiently discriminated aggressive HB in a training set of 19 HBs and a test set of 46 HBs. In fact, this miR signature was associated with invasive, poorly differentiated, and metastatic HB tumors [24].

Overexpression of the oncogenic transcription factor PLAG1 is a frequent alteration in HB [52]. MiR-492 has been proposed as a potential biomarker in metastatic HB (Table 2). This miR was identified to be located within the HB marker gene keratin 19 (*KRT19*), a marker for hepatic progenitors and cancer stem cells, previously related to the metastatic progression of HCC [53,54]. PLAG1 might co-regulate the expression of KRT19 and the miR-492 release. Significantly higher levels of co-expressed KRT19 and miR-492 were found in metastatic HB tumor samples, suggesting their role in the progression of this disease [32]. Recently, the same group has reported that miR-492 overexpression correlates with metastatic disease and high-risk tumors and determines reduced event-free survival in a cohort of 44 HB patients [33]. All the reported miRNAs with clinical value as biomarkers in HB have been included in Table 2.

Another study analyzed miR expression in the main HB epithelial subtypes, revealing different miR expression patterns in fetal and embryonal HB. The authors observed higher levels of miR-18a in embryonal compared to fetal samples, but histological classification did not correlate with survival. In HB embryonal samples, lower miR-122 levels were observed compared to liver normal tissue, whereas higher miR-221 and lower miR-17-5p, miR-195, miR-210, and miR-214 were detected in HB fetal samples. Of note, low miR-222 and miR-224, and high miR-21 independently predicted larger overall survival in that HB cohort. However, the small number of HB samples included (*n* = 23) and the fact that only 14 miRs were measured are important limitations of this study [47]. Conversely, if we analyze the miR expression patterns during liver development, we surprisingly observed an inverse correlation regarding miR expressions in embryonic and fetal HB. Thus, miR-18a is highly expressed in adult compared to embryonic liver, and miR-122 is overexpressed in embryonic and fetal compared to the adult liver [55]. Of note, a recent study in a cohort of 22 HB patients showed that expression levels of miR-17 were significantly lower in tumor samples and correlate with worse prognosis. In this work, miR-19b was found overexpressed in the embryonal subtype compared to fetal subtype [56]. In concordance with these results, both exosomal and plasma in miR-21 were found significantly higher in a cohort of 32 HB samples compared with a control group. Vascular invasion, PRETEXT staging system, tumor metastases, and exosomal miR-21 were independent risk factors that could affect the event-free survival of HB patients. Moreover, it was found that exosomal miR-21 was significantly more accurate as compared with the alpha-fetoprotein (AFP) levels in diagnosing HB [57].

Interestingly, a larger study including 76 HB patients identified that combined low miR-34a/b/c expression independently predicts poor prognosis [58]. The same group confirmed these results using an exosomal miR-34a/b/c panel in an independent cohort of 89 HB patients, observing that the prognostic value of a panel of serum exosomal miRNAs including miR-34a/b/c was superior to other risk factors such as AFP. Although this miRNA panel also showed diagnostic value, in this case, there were no differences compared to AFP levels [59]. Moreover, miR-4510 was found downregulated in HB tumors compared to adjacent normal liver samples, probably based on its tumor suppressor role as an inhibitor of the Wnt/β-Catenin pathway [25]. Finally, a study involving the comparison between three independent platforms (next-generation sequencing, microarray, and NanoString) in 12 formalin fixed paraffin embedded (FFPE) samples from HB patients identified an alternative pattern in a poorly-differentiated HB with an aggressive phenotype [60,61].

## 5. Conclusions

New biological studies involving miRs could serve to identify novel effective biomarkers with prognostic value that would be helpful for risk stratification as well as provide alternative therapeutic strategies for the treatment of HB, especially in those patients with advanced tumors or metastatic disease. Thus, the identification of promising candidates for miR replacement therapy may represent a very interesting therapeutic solution. However, most of the studies involving miRNA in HB have been performed using a relatively small number of samples, and in some cases, they have included only a group of candidate miRNAs rather than a global analysis.

Therefore, further validation using large patient cohorts and preclinical models such as HB patient-derived xenografts is required and would highlight which of these progressive findings involving miRs will be extended to future clinical studies.

## Figures and Tables

**Table 1:** MicroRNAs with potential therapeutic value in Hepatoblastoma.

MicroRNA	Role	Therapeutic Impact	References
miR-100/let-7a-2/miR-125b-1 cluster	Tumor supressor	In vivo cooperation. OE of the first cluster concomitant with inhibition of the second one blocked tumor formation	Cairo et al., 2010 [24]
miR-371 cluster	Oncogenic		
miR-122	Tumor supressor	Sensitizes to 5-FU via Bcl-2 and Bcl-XL DR and P53 activation	Yin et al., 2011 [45]
miR-214	Tumor supressor	Sensitizes to gemcitabine via UCP2 targeting	Yu et al., 2016 [46]
miR-624-5p	Tumor supressor	Targets β-catenin and impairs tumor growth in vivo	Indersie et al., 2017 [26]
miR-4510	Tumor supressor	Decreases proliferation and induces apoptosis in vitro through Wnt/β-catenin inhibition	Cartier et al., 2017 [25]
miR-34a-5p	Tumor supressor	Reduces tumor growth and microvascular density	Dong et al., 2016 [38]
miR-125b	Tumor supressor	Decreases cell growth, migration and invasion	Pei et al., 2016 [31]
miR-492	Oncogenic	Targets CD44 and enhances anchorage-independent growth, migration and invasion	von Frowein et al., 2018 [33]
miR-378a	Tumor supressor	Sensitizes to sorafenib and inhibits cell proliferation and invasion	Fu et al., 2018 [48]
miR-26a-5p	Tumor supressor	Inhibits proliferation and colony formation	Zhang et al., 2018 [37]
miR-1250-3p	Tumor supressor	Decreases cell growth and invasion	Liu et al., 2018 [49]

OE, Overexpression; DR, downregulation.

**Table 2:** MicroRNAs as prognostic markers in Hepatoblastoma.

MicroRNA	Expression	Clinical Significance	References
miR-492	High	Metastatic disease	[30]
miR-21	High	Independent markers of increased survival	[41]
miR-222 and miR-224	Low		
Combined miR-34a/b/c	Low	Independent unfavourable prognostic factor	[53]
Exosomal miR-21	High	Independent predictor of larger EFS ^1^	[52]
Exosomal miR-34a/b/c	Low	Independent unfavourable prognostic factor	[54]
miR-492	High	Correlation with aggressive tumors and reduced EFS ^1^	[31]
miR-17	Low	Worse prognosis	[51]

^1^ Event-free Survival.
